# Successful Laparoscopic Oviductal Artificial Insemination in the Endangered Tsushima Leopard Cat (*Prionailurus bengalensis euptilurus*)

**DOI:** 10.3390/ani12060777

**Published:** 2022-03-19

**Authors:** Akinori Azumano, Miya Ueda, Mika Nomura, Masashi Usui, Midori Ichinose, Yojiro Yanagawa, Satoshi Kusuda, Yuki Matsumoto, Koichi Murata

**Affiliations:** 1 Yokohama Zoological Gardens, Yokohama 241-0001, Japan; ueda.m@hama-midorinokyokai.or.jp (M.U.); nomura.104@hama-midorinokyokai.or.jp (M.N.); usui@hama-midorinokyokai.or.jp (M.U.); haemoproteus@gmail.com (K.M.); 2 Preservation and Research Center, Yokohama 241-0804, Japan; mi01-ichinose@city.yokohama.lg.jp; 3 Faculty of Veterinary Medicine, Hokkaido University, Sapporo 060-0818, Japan; yoji-y@vetmed.hokudai.ac.jp; 4 Faculty of Applied Biological Sciences, Gifu University, Gifu 501-1193, Japan; kusuda@gifu-u.ac.jp; 5 Anicom Specialty Medical Institute Inc., Yokohama 231-0033, Japan; yuki.matsumoto@ani-com.com; 6 Anicom Insurance, Inc., Tokyo 171-0033, Japan; 7 College of Bioresource Science, Nihon University, Fujisawa 252-0880, Japan

**Keywords:** Tsushima leopard cat, ex situ conservation, laparoscopic oviductal artificial insemination, equine chorionic gonadotropin, porcine luteinizing hormone

## Abstract

**Simple Summary:**

The Tsushima leopard cat is an endangered wild felid that lives solely on Tsushima Island, Nagasaki, Japan. Approximately, only 100 Tsushima leopard cats can be found in the wild, and there are concerns that the population will further reduce due to habitat degradation and traffic accidents. In 1994, Japan’s Ministry of the Environment (MOE) developed a conservation and breeding project for the Tsushima leopard cat. The MOE is working with the Japanese Association of Zoo and Aquariums for ex situ conservation of this species. However, considering genetic diversity, it is difficult to conduct captive breeding programs using only natural breeding; hence, assisted reproductive technologies are required. This study aimed to breed Tsushima leopard cats using artificial insemination (AI) by depositing sperms into the oviducts. Ovulation was artificially induced in two females, laparoscopically inseminated with fresh sperms into the oviducts. The pregnancies were monitored via fecal levels of progestogens and radiography. One female had spontaneous delivery of a female kitten 66 days post-AI. This is the first successful case of AI in a Tsushima leopard cat.

**Abstract:**

The Tsushima leopard cat (*Prionailurus bengalensis euptilurus*) is an endangered wild felid that lives solely on Tsushima Island, Nagasaki, Japan. Japan’s Ministry of the Environment is working with the Japanese Association of Zoo and Aquariums for ex situ conservation of this species. However, considering genetic diversity, it is difficult to conduct captive breeding programs by natural breeding alone; hence, assisted reproductive technologies are required. This study aimed to breed Tsushima leopard cats, which otherwise cannot be paired, using laparoscopic oviductal artificial insemination (AI). Female Tsushima leopard cats (female 1, aged 7 years; female 2, aged 6 years) were treated with 200 IU equine chorionic gonadotropin, followed by administration of 1000 IU porcine luteinizing hormone (pLH) after 96 h to induce ovulation. Laparoscopic AI was performed 32 h post-pLH administration. Females 1 and 2 were inseminated in the oviduct with 2.4 × 10⁶ and 3.3 × 10⁶ motile spermatozoa, respectively, collected from two males. Pregnancy was confirmed by radiography 45 and 51 days post-AI in females 1 and 2, respectively; one fetus was found in female 2. Moreover, female 2 had spontaneous delivery of a female kitten 66 days post-AI. This is the first successful case of AI in a Tsushima leopard cat.

## 1. Introduction

The Tsushima leopard cat (*Prionailurus bengalensis euptilurus*), a small wild felid, is a subspecies of the leopard cat (*Prionailurus bengalensis*) [1] that inhabits solely Tsushima Island, Nagasaki, Japan (129° E, 34° N). There are approximately only 100 Tsushima leopard cats found in the wild, and there are concerns that the population will further decline owing to habitat degradation and traffic accidents [2]. Therefore, in 1994, Japan’s Ministry of the Environment (MOE) declared the Tsushima leopard cat as a nationally endangered species of wild fauna and flora and formulated a plan for the rehabilitation of its natural habitats and the maintenance of viable populations. As a part of this effort, in 1999, the MOE collaborated with the Japanese Association of Zoos and Aquariums to initiate captive breeding using wild individuals as the starting point for ex situ conservation. Thus, captive breeding was successfully achieved for the first time in 2000 [3]. As of 2020, 32 animals were being bred at nine facilities in Japan. However, in recent years, the captive population has been declining and aging, and genetic diversity is being lost because of natural breeding of specific pairs; therefore, a new, long-term captive breeding program for the conservation of this rare species is urgently needed. However, pairing of this species is difficult because of incompatibility between individuals, and the high aggression of males towards females during mating makes it difficult to implement a captive breeding program that fosters genetic diversity.

To address this problem, artificial reproduction using assisted reproductive technologies (ARTs) has been considered; however, previous attempts at artificial insemination (AI) have failed, which could be due to a low sperm number collected from the males. In felids, transcervical and laparoscopic, intrauterine AI are considered effective to some extent [4,5,6,7,8,9,10,11,12,13,14,15,16]; however, these methods require a relatively large number of sperm, while oviductal AI requires a smaller number of sperm for conception [17,18,19,20]. Hence, oviductal AI is a better method when the number of available sperms is low.

This study aimed to perform laparoscopic oviductal AI in two female non-breeding Tsushima leopard cats using fresh semen to produce offspring and to investigate the potential for achieving conception.

## 2. Materials and Methods

The animal study protocol was approved by the Animal Care and Use Committee of Hokkaido University (22-0019). All procedures were conducted in accordance with the Guideline for Proper Conduct of Animal Experiments, Science Council of Japan (2006). Every effort was made to ensure the welfare of the animals and reduce their stress.

### 2.1. Animals and Husbandry

Two females (female 1, aged 7 years; female 2, aged 6 years) and two males (male 1, aged 11 years; male 2, aged 2 years) Tsushima leopard cats housed at the Yokohama Zoo (1175-1 Kamishirane-cho, Asahi-ku, Yokohama, Japan) were included. The two females had a history of pregnancy (female 1: kittens were eaten by the dam immediately after birth in 2016, 2017, and 2018; female 2: a kitten died two days after birth in 2017), whereas the two males had no history of breeding. The weights of female 1, female 2, male 1, and male 2 before AI were 4.4 kg, 3.6 kg, 3.6 kg, and 4.6 kg, respectively. The four animals were separately housed in an indoor/outdoor facility (2.0 m × 6.3 m × 2.6 m) with natural light and indoor heating of approximately 15 °C. They were fed horse meat, chicken meat, chicken heads, and mice and had free access to water.

### 2.2. Behavioral Observations

Each female’s room had closed-circuit television for surveillance of estrus behavior, and 24 h behavioral observations were conducted throughout December to April. Estrous behaviors were defined as rubbing and rolling, restlessness, and calling.

### 2.3. Fecal Hormone Analysis

Fresh-looking feces (except for those excreted in water) were collected once daily (between 9:00 a.m. and 12:00 noon) and analyzed from 20 days before to 80 days after AI. Feces were stored at −20 °C immediately after collection. Frozen fecal samples were dried in a forced convection oven (FC-410: Advantec, Tokyo, Japan) at 100 °C for approximately 6 h, crushed using a hammer, and pooled. A total of 0.1 g of the fecal powder was extracted in 5 mL of 80% methanol by vortex-mixing for 30 min, as reported previously [21]. After centrifugation (1444× *g* for 10 min), the supernatant methanol fraction was removed and diluted (at 1:10 for the estradiol-17β [E2] assay and 1:100 or 1:1000 for progesterone [P4] assay) using assay buffer (phosphate buffer containing 0.1% bovine serum albumin). The dilutions were determined to be within the range of concentrations of the standard curves for each hormone. Fecal concentrations of E2 and P4 were determined by enzyme immunoassays (EIAs) used on the Tsushima leopard cat in a previous report [21], using E2 antiserum (1:3,000,000; FKA 236-E; Cosmo Bio, Tokyo, Japan) and P4 antiserum (1:400,000; AF17091287-001; consignment of production to Cosmo Bio). Serial dilutions of Tsushima leopard cat fecal extracts showed parallelism to the E2 and P4 standard (E2, 1:10–1:640, R2 = 0.9622; P4, 1:100–1:6400, R2 = 0.9896). Recovery of amounts of E2 or P4 added to a pool of diluted fecal extracts was 105.4 and 100.7%, respectively. Intra- and inter-assay coefficients of variation were 6.8% and 15.6% for E2, 6.8% and 12.7% for P4, respectively.

E2 and P4 baseline concentrations were calculated for each female using an iterative process that excluded values exceeding the mean + 1.5 standard deviation (SD) and the mean + 1.2 SD, respectively [22]. For E2, values greater than two times the baseline value were considered “elevated”. For P4, values greater than three times the baseline value were considered “elevated”. Ovulation was presumed to have occurred when the progesterone concentration was more than three times higher than the baseline value and the increase lasted for at least one week.

### 2.4. Induction of Ovarian Activity

Females were intramuscularly administered 200 IU equine chorionic gonadotropin (eCG) (ASKA Animal Health, Tokyo, Japan) to stimulate follicle development. At 96 h post-eCG administration, 1000 IU porcine luteinizing hormone (pLH) (Ray Biotech, Norcross, GA, USA) was intramuscularly administered to induce ovulation. Exogenous gonadotropin administration was performed while the animals were manually restrained with gloved hands.

### 2.5. Semen Collection and Processing

Semen collection was performed under anesthesia. Males were immobilized using 0.04 mg/kg medetomidine (Kyoritsu Seiyaku, Tokyo, Japan) and 2.0 mg/kg ketamine (Fujita Pharmaceutical, Tokyo, Japan); and after tracheal intubation, anesthesia was maintained by inhalation of 1–2% isoflurane (MSD Animal Health, Tokyo, Japan). Partial reversal was performed with 0.12 mg/kg atipamezole (Kyoritsu Seiyaku) at the end of the anesthesia.

Semen was collected by a standardized method of urethral catheterization and elec-tro-ejaculation [23,24]. Before semen collection, the pubic region was cleaned, and the pe-nis was washed with sterile physiological saline. Catheterization (~8 cm) using a 3Fr feeding tube (Atom Medical, Tokyo, Japan) was initiated 20 min after medetomidine treatment. The catheter was placed in the urethra for 1 min to collect semen. After catheterization, urine was removed using a urethral catheter. Subsequently, electro-ejaculation was performed. Semen was collected by inserting a 0.95 cm diameter electro-ejaculator probe used for cats and dogs (Minitüb GmbH, Tiefenbach, Germany) followed by 80 stimulations that were conducted in three series with 30 simulations (2 V to 4 V) in the first one, 30 stimulations (3 V to 5 V) in the second one, and 20 stimulations (4 V to 5 V) in the final one. Sperms were collected in sterile 1.5 mL plastic conical tubes.

Since the volume of each semen sample collected was small, they were pooled and evaluated under a microscope for motility and progressive motility. Motility was visually evaluated by a researcher. The sperm count was assessed using a hemocytometer chamber. Viability and abnormality were also assessed using eosin–nigrosine staining [25]. Semen was centrifuged (300× *g* for 6 min) to remove seminal plasma, diluted 1:4 in HEPES-buffered feline-optimized culture medium (FOCMH) [26] (Table 1), and stored at 30 °C in the dark until AI. Immediately before AI, spermatozoa from two males were mixed and centrifuged (300× *g* for 6 min) to remove the supernatant, resuspended in 20 μL of FOCMH, and deposited into the left and right oviducts of the females.

### 2.6. Vaginal Smear and Ovarian Assessment

Vaginal smear and ovarian assessments were performed immediately before AI. For the vaginal smear test, vaginal epithelial cells were collected with a cotton swab moistened with saline and smeared on glass slides. The glass slides were stained with Hemacolor^®^ (Merck, Darmstadt, Germany) and observed under a microscope. Ovarian assessment was performed by direct observation of the ovaries using a 7 mm diameter laparoscope (Olympus Medical Systems, Tokyo, Japan).

### 2.7. Laparoscopic AI

**Figure 1:**
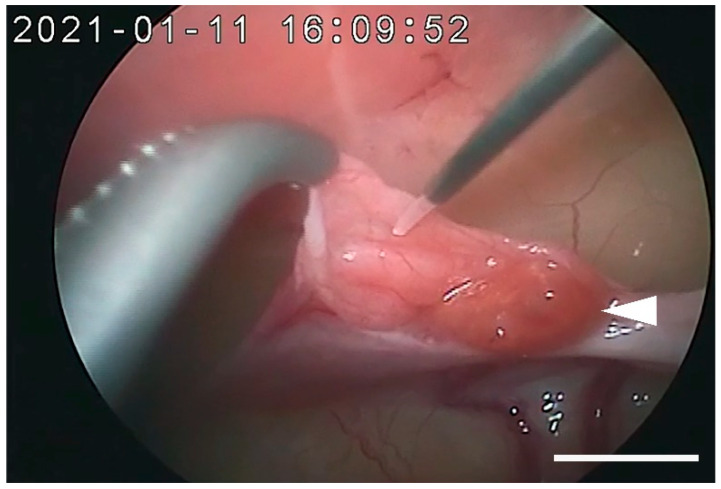
Laparoscopic view of intraoviductal artificial insemination (AI) using the AI needle. The white arrow indicates the position of the left ovary. Bar = 10 mm.

AI was conducted under anesthesia at 32 h post-pLH administration on 28 December 2020 (female 1) and 11 January 2021 (female 2). Females were immobilized in an anesthesia box using isoflurane, and anesthesia was maintained by isoflurane inhalation after tracheal intubation. They were placed resting on their backs and held high on their tails via a surgical tilt table tilted at 15°. The abdomen was sheared and disinfected similar to sterilization surgery. Two trocar cannulas for laparoscope and forceps were placed at 1 cm cranial and 4 cm caudal to the umbilicus. Laparoscopic oviductal AI was performed by modifying a previously reported method [20]. The AI needle consists of an outer tube, which is a 90 mm long, blunted 20-gauge spinal needle (Terumo, Tokyo, Japan) and an inner tube with polyethylene tubing (PE-10; Intramedic; Becton Dickinson and Company, Sparks, MD, USA) attached to a 27-gauge needle (Terumo). The inner tube was approximately 1 cm longer than the outer tube and it protruded at one end (needle tip) to prevent mucosa damage of the oviduct. The AI needle was connected to a 1 mL syringe and the tip of the AI needle was pre-filled with 10 µL of resuspended semen. A 16-gauge polypropylene IV catheter (Terumo) was percutaneously inserted near the ovary, and an AI needle was inserted intraperitoneally through the polypropylene IV catheter. The edge of the ovarian bursa was grasped with Maryland forceps (A64320A; Olympus Medical Systems), the oviductal ostium was exposed and visualized, the tip of the AI needle was inserted 1 cm into the oviduct, and semen was deposited (Figure 1).

### 2.8. Pregnancy Diagnosis and Observation of Delivery

Pregnancy was diagnosed approximately 50 days post-AI. Radiography was used to confirm the presence and number of fetuses. Females were manually restrained with gloved hands. We also used closed-circuit television to monitor the activities of pregnant females until delivery.

### 2.9. Genetic Testing

Owing to the low genetic diversity of Tsushima leopard cats [27], existing microsatellite markers could not be used. Thus, we used whole-genome sequencing for paternity testing. The total DNA of four cats (female 1, male 1, male 2, and kitten) was extracted from blood samples using a DNeasy Blood & Tissue Kit (Qiagen, Venlo, The Netherlands). The sequencing library was generated using a TruSeq DNA PCR-Free Kit (Illumina, San Diego, CA, USA) and sequenced using NovaSeq 6000 (Illumina). Low-quality reads (phred score < 30), adapter sequences, and reads with lengths <50 nucleotides were removed using Trim-galore [28]. The quality-filtered reads were mapped and aligned to the domestic cat genome (felCat9) [29] by dynamic read analysis for genomics (DRAGEN v3.7.5, Illumina). DRAGEN provided a gVCF file using the enable-map-align option. Four gVCF files were merged into one using the DRAGEN --enable-joint-genotyper option. After calling variants with gVCF, we extracted single nucleotide polymorphisms (SNPs) for subsequent analysis. Quality control for SNPs was performed using PLINK software v. 1.90 [30]. The missing rates for each locus (--geno option) was 0%.

For allele frequency-based quality control, SNPs with a minor allele frequency of <0.125 indicated that at least one allele was polymorphic between eight individuals. We used kinship and the proportion of SNPs with zero identical-by-state (IBS0) to estimate the degree of relatedness and paternity testing. Kinship and IBS0 were calculated using the --make-king-table option implemented in PLINK software v2.00a2LM [31].

## 3. Results

### 3.1. Behavioral Observation

No estrous behaviors were observed from December to January in either female.

### 3.2. Fecal Hormone Analysis

**Figure 2:**
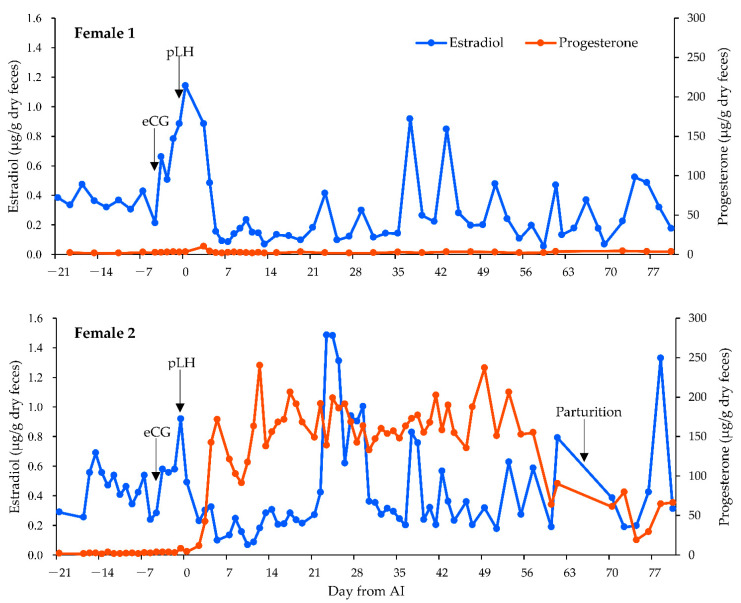
Fecal hormone concentrations of the two female Tsushima leopard cats studied. The blue line represents estradiol-17β (E2), whereas the orange line represents progesterone (P4). Day 0 indicates the day of artificial insemination (AI). The day of administration of each equine chorionic gonadotropin (eCG) and porcine luteinizing hormone (pLH) are noted for each female.

The E2 baseline concentrations for females 1 and 2 were 0.13 μg/g dry feces and 0.23 μg/g dry feces, respectively. In female 1, fecal E2 concentrations increased after eCG administration and peaked on the same day as AI. In female 2, fecal E2 concentrations increased after eCG administration and peaked on the same day as pLH administration. The P4 baseline concentrations for females 1 and 2 were 1.5 μg/g dry feces and 1.4 μg/g dry feces, respectively. In female 1, fecal P4 concentrations remained close to baseline after AI. The average P4 concentration for the first 30 days post-AI was 2.7 μg/g dry feces. In female 2, fecal P4 concentrations increased from baseline after AI, which suggested that ovulation had occurred based on sustained elevations in fecal P4 concentrations. The average P4 concentration for the first 30 days post-AI was 150.5 μg/g dry feces. Fecal P4 concentrations remained high until delivery (Figure 2).

### 3.3. Semen Characteristics

On the day of each female’s AI, sperm was collected from males 1 and 2 and pooled (Table 2). Female 1 received 2.4 × 10^6^ total motile sperm, whereas female 2 received 3.3 × 10^6^ total motile sperm. All sperm was inseminated into the females’ oviducts.

### 3.4. Vaginal Smear and Ovarian Assessment

**Figure 3:**
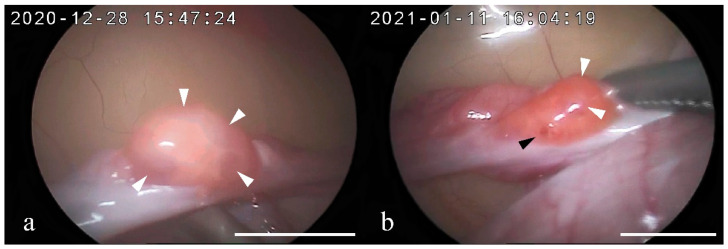
Representative pictures of the ovary observed through laparoscopy in the two Tsushima leopard cats after stimulation with 200 IU equine chorionic gonadotropin followed by 1000 IU porcine luteinizing hormone (pLH) 96 h later. Observations were made 32 h post-pLH administration: (**a**) female 1, right ovary; (**b**) female 2, left ovary. White arrow and black arrow indicate follicles (diameter, ≥2 mm) and corpus hemorrhagicum, respectively. Bar = 10 mm.

Keratinized epithelial cells were observed in both females 1 and 2 using a vaginal smear test immediately before AI. Observation of the ovaries by laparoscopy showed developed follicles in the right and left ovaries of both females 1 and 2. In female 1, four follicles ≥2 mm in diameter were found in the right ovary and three in the left ovary. In female 2, four follicles ≥2 mm in diameter were found in the right ovary and four follicles and one corpus hemorrhagicum (CH) in the left ovary (Figure 3).

### 3.5. Pregnancy Diagnosis and Observation of Delivery

**Figure 4:**
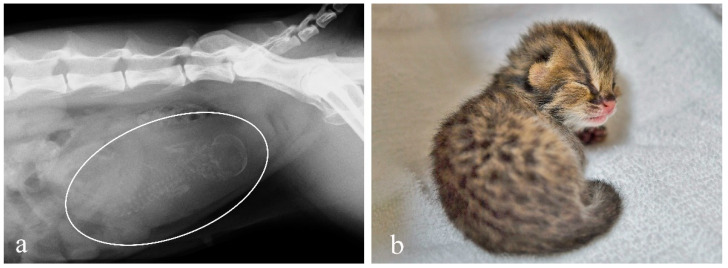
(**a**) Pregnancy diagnosis by radiography 51 days post-AI is confirmed by mineralized skeleton of a fetus (white circle). (**b**) Image of the Tsushima leopard cat kitten two days after birth.

Pregnancy diagnosis was performed 45 days post-AI for female 1 and 51 days post-AI for female 2. Radiography of female 2 showed mineralized skeletons of a fetus. On day 66 post-AI, a healthy female kitten was delivered spontaneously by female 2 (Figure 4).

### 3.6. Genetic Testing

The kinship ranged from −0.09 to 0.23 for the six analysis pairs based on 4,640,102 genome-wide SNPs (Table 3). A kinship value between 0.177 and 0.354 indicated a first-degree relationship, i.e., parent–offspring or full sibling relationship [31]. The range of the first-degree relationship included two pairs (kitten and female 2; kitten and male 2). The kinship between the kitten and male 2 was 0.23, which was approximately the same value as that between the kitten and female 2, and IBS0 was close to 0 (i.e., 0.01). In contrast, the kinship between the kitten and male 1 was −0.09 (IBS0 = 0.11), indicating biologically distant relationships. These results indicate that male 2 is the biological father of the kitten.

## 4. Discussion

To our knowledge, this is the first report of a successful AI in the Tsushima leopard cat. For ex situ conservation of wildlife, AI is an effective option for captive breeding programs because it allows the breeding of individuals that are difficult to pair and the use of semen collected from wild individuals [32,33].

Reproductive seasonality can complicate treatment with exogenous gonadotropins and scheduling of AI. Domestic cats are seasonal breeders, making it difficult to maintain pregnancy with AI during the non-breeding season because the embryos may fail to develop normally or the corpora lutea may regress earlier than in the natural course of pregnancy [34]. As the Tsushima leopard cat is also a seasonal breeder, it is possible that pregnancy maintenance is difficult during the non-breeding season. A previous study in the Amur leopard cat (*Prionailurus bengalensis euptilurus*) reported that embryo death occurred with AI conducted in the non-breeding season (November), although the cause was not identified [35]. Therefore, it may not be appropriate to conduct AI during the non-breeding season. In contrast, exogenous gonadotropins are generally administered in reproductively quiescent females because spontaneous ovulation and the presence of active luteal tissue on the ovary during the breeding season can profoundly affect ovarian responses to exogenous gonadotropin treatment [36]. The breeding season of the Tsushima leopard cat is from January to March [3]. Hence, our strategy was to conduct AI during the pre-breeding season (December) to the early breeding season (January) when pregnancy maintenance was expected and ovarian activity was less pronounced. Swanson et al. reported that altrenogest pretreatment was effective for ovarian suppression before exogenous gonadotropin treatment in the AI of small wild felids [19,20]. The use of altrenogest as a pretreatment to conduct AI during the breeding season using a fixed-time approach in the Tsushima leopard cat should be examined in further studies.

Since estrus behavior was not observed before exogenous gonadotropin administration, and the fecal P4 concentration was at baseline before AI, it was believed that there was no possible ovarian activity that could interfere with the action of exogenous gonadotropin. Thus, the timing of exogenous gonadotropin administration initiation was considered appropriate for both females 1 and 2. In this study, the doses of eCG and pLH were 200 IU and 1000 IU, respectively, which are similar to those used in AI studies in the Pallas’s cat (*Otocolobus manul*) [19] and clouded leopards (*Neofelis nebulosa*) [37]. Laparoscopic observation of the ovaries showed that the eCG/pLH dose caused follicular development in both females and ovulation in at least one female, although it was unclear whether the doses were appropriate. In this study, the administration interval between eCG and pLH was 96 h, whereas in many previous studies of AI in wild felids, intervals of 80–85 h were reported [6,7,8,9,10,11,12,13,18]. In contrast, in the laparotomic intrauterine AI of the Amur leopard cat, human chorionic gonadotropin was administered 120 h post-eCG administration [35]. Moreover, for transcervical AI in the Asiatic golden cat (*Catopuma temmincki*), ovulation induction by gonadotropin-releasing hormone analogues was performed at approximately 96 h after the onset of estrus [15]. In this study, pLH was administered at approximately the peak fecal E2 concentration in both females 1 and 2. Considering the time required for E2 metabolism and excretion into the feces, the interval of administration was considered ideal. However, further studies on the appropriate dose and administration intervals of exogenous gonadotropins are warranted.

Sperm collection was performed twice for each male. The average total sperm count and motility were 3.8 × 10^6^ and 33.8%, respectively. In this study, the total sperm count and motility of Tsushima leopard cat semen were much lower than those reported for Amur leopard cat semen (mean total sperm count: 11.4 × 10^6^; mean motility: 84%), although they belong to the same subspecies [38]. Previous studies of domestic cats have shown that the sperm count required for fertilization by intravaginal and intrauterine AI is 80 × 10^6^ and 8 × 10^6^, respectively [39,40]. The age of the males, male 1 being older than male 2, may have affected the outcome [41], but the results show the collection of sufficient sperm volume for vaginal and intrauterine AI of the Tsushima leopard cat to be a challenge.

The results of the vaginal smear test indicated that exogenous gonadotropin successfully induced follicular development, consistent with observation results of the ovaries during laparoscopy. In female 1, the fecal P4 concentration remained at baseline even after AI, suggesting that she failed to ovulate. Previous studies on the Pallas’s cat reported that exogenous gonadotropin treatment during the pre-breeding season resulted in the failure to achieve a normal ovarian response [42]. Thus, we speculate that because ovulation induction was performed prior to the breeding season in female 1, it resulted in failure; however, it is unclear why ovulation failed to occur. In female 2, laparoscopic observation of the ovaries revealed a single CH in the left ovary. Ovulation was confirmed by observation of the ovaries at 32 h post-pLH administration, indicating that the time to ovulation after pLH administration in the Tsushima leopard cat was comparable to that of domestic cats [17].

## 5. Conclusions

Our study demonstrated that it is possible to obtain offspring using laparoscopic oviductal AI in Tsushima leopard cats. In this study, 3.3 × 10^6^ motile spermatozoa were inseminated laparoscopically into the oviduct of one female to obtain a kitten. In other wild felid species, such as the Amur tiger (*Panthera tigris altaica*) [18], the Pallas’s cat (*Otocolobus manul*) [19], the ocelot (*Leopardus pardalis*) [20], and the fishing cat (*Prionailurus viverrinus*) [20], laparoscopic oviductal AI has been successfully achieved with <5 × 10^6^ motile sperms. Thus, it was suggested that even in Tsushima leopard cats, kittens could be obtained by depositing a small amount of sperm into the oviduct, as with these wild felids. Therefore, laparoscopic oviductal AI may be a useful method for conservation of the Tsushima leopard cat. However, as this study only included a very small sample size, only one successful case of AI was reported. Thus, it is necessary to conduct additional research on the appropriate dose and administration interval of exogenous gonadotropins, the success rate of laparoscopic oviductal AI, and the timing of AI implementation to improve the feasibility of breeding programs through the introduction of ARTs.

## Figures and Tables

**Table 1:** Composition of HEPES-buffered feline-optimized culture medium.

Component	Concentration (mM)
NaCl	100.0
KCl	8.0
KH_2_PO_4_	1.0
CaCl_2_-2H_2_O	2.0
MgSO_4_-7H_2_O	1.0
Glucose	3.0
L-Lactate	6.0
Pyruvate	0.1
NaHCO_3_	5.0
HEPES	20.0
Glutamine	1.0
Taurine	0.1
NEAA	×1
BSA ^1^	4.0 mg/mL

Ref. [26] Herrick et al. 2007 Biology of Reproduction, 76: 858–870 ^1^ Fraction V.

**Table 2:** Sperm quality collected from the two male Tsushima leopard cats.

Parameter	Date of Semen Collection			
	28 December 2020		11 January 2021	
	Male 1	Male 2	Male 1	Male 2
Volume (μL) ^1^	8 + 60	7 + 50	10 + 25	10 + 35
Concentration (×10⁶/mL)	45	77	84	129
Total sperm (×10⁶)	2.7	3.9	2.9	5.8
Motility (%)	30	40	15	50
Progressive motility (%)	15	20	5	30
Viability (%)	– ^2^	–	–	61
Abnormality (%)	–	–	–	47

^1^ The volume represents the sum of the semen volumes collected by the two methods. The left value is the volume of semen collected by urethral catheterization, and the right value is that collected by electro-ejaculation. ^2^ Not examined due to low volume and total sperm count.

**Table 3:** Genetic relatedness based on 4,640,102 single-nucleotide polymorphisms.

ID1	ID2	IBS0	Kinship
Kitten	Female 2	0.01	0.22
Kitten	Male 1	0.11	−0.09
Kitten	Male 2	0.01	0.23
Male 1	Female 2	0.09	0.04
Male 2	Female 2	0.05	0.13
Male 2	Male 1	0.08	0.05
